# Possible effects of royal jelly against neuronal injury in the hippocampus of ovariectomized rats with pentylenetetrazol-induced seizures: Role of luteinizing and follicle-stimulating hormones

**DOI:** 10.22038/AJP.2024.25151

**Published:** 2025

**Authors:** Asma Momeni, Mohammad Reza Salahshoor, Mohammadreza Afarinesh, Cyrus Jalili

**Affiliations:** 1 *Department of Anatomical Sciences, School of Medicine, Hormozgan University of Medical Sciences, Bandar abbas, Iran*; 2 *Department of Anatomical Sciences, School of Medicine, Kermanshah University of Medical Sciences, Kermanshah, Iran*; 3 *Fertility and Infertility Research* *‏ ‏* *Center, Kermanshah University of* *‏ ‏* *Medical Sciences, * *‎* *Kermanshah* *‎* *, Iran*; 4 *Neuroscience Research Center, Institute of Neuropharmacology, Kerman University of Medical Sciences, Kerman, Iran*

**Keywords:** Menopause, Neurons, Ovariectomy, Pentylenetetrazol, Royal jelly, Seizures

## Abstract

**Objective:**

This study aimed to investigate the potential impact of royal jelly (RJ) on hippocampal neurons in an ovariectomized (OVX) rat model with pentylenetetrazol (PTZ)-induced seizures by assessing luteinizing (LH) and follicle-stimulating (FSH) hormones.

**Materials and Methods:**

Fifty-six female rats (n=7/group) were divided into groups receiving saline (CTL, OVX, RJ, and OVX-RJ) and those undergoing PTZ-induced seizures (PTZ, PTZ-OVX, PTZ-RJ, and PTZ-OVX-RJ). OVX rats underwent bilateral ovary removal, followed by a 15-day RJ treatment at 300 mg/kg. The seizure model commenced 24 hours after the final RJ dose. Serum LH and FSH levels were measured, and Golgi staining assessed hippocampal neuron morphology.

**Results:**

The RJ group exhibited elevated LH and FSH levels compared to CTL. However, the PTZ-RJ group showed no significant changes in these hormones relative to the PTZ and CTL groups. In OVX-RJ rats, LH and FSH levels decreased compared to the RJ group, while PTZ-OVX-RJ rats showed increased levels. Dendritic spines remained unchanged in both the RJ and PTZ-RJ groups compared to the CTL and PTZ groups, respectively. Notably, OVX-RJ exhibited reduced spines compared to the RJ group, while PTZ-OVX-RJ showed an increase.

**Conclusion:**

RJ may protect against estrogen deficiency and seizure-related adverse effects on hippocampal neurons in OVX rats, highlighting its potential as a beneficial dietary supplement.

## Introduction

Epilepsy is the fourth most common neurological disorder, affecting approximately 65 million people worldwide. Important risk factors for developing epilepsy mainly fall into two categories: genetic and acquired; however, in some cases, the cause is unknown (England et al., 2012). Neuronal death, especially in the hippocampus, is a common feature of acquired epilepsy and is thought to contribute to cognitive dysfunction (Pitkänen and Sutula, 2002). Epileptogenesis is associated with subtle neuronal injury, gliosis and microgliosis, as well as increasingly intense and persistent inflammatory states in the neuronal tissue microenvironment (Alyu and Dikmen, 2017; Pearson-Smith and Patel, 2017).

Oxidative stress plays an important role in hereditary and acquired epilepsy. Oxidative damage occurs when reactive oxygen species production exceeds the detoxification capacity of endogenous antioxidants   (Pearson-Smith and Patel, 2017). Treatment with various compounds that reduce oxidative stress (antioxidants, NADPH oxidase inhibitors, etc.) has been shown to prevent seizure-induced neuronal cell death (Frantseva et al., 2000). The use of drugs to reduce brain inflammation to treat epilepsy and seizures has received increasing attention in recent years. There is evidence of a link between brain inflammation and the onset and exacerbation of epilepsy. Naturally, occurring antioxidants and anti-inflammatory agents are probably preferred over them, as some synthetic antioxidants have been reported to have side effects (Osuntoki and Korie, 2010). Royal jelly (RJ) is a thick substance produced by the hypopharyngeal and mandibular glands of worker bees (*Apis mellifera*) and serves as vital nourishment for the queen bee larvae and the queen bee herself (Narita et al., 2006). Phenolic compounds are commonly present in RJ as flavonoids which contribute to the functional properties of bee products, including antioxidants, antibacterial, and anti-inflammatory effects, and protection against cell apoptosis (Karadeniz et al., 2011). RJ is composed of water (50–60%), protein (18%), carbohydrates (15%), lipids (3–6%), mineral salts (1.5%), and vitamins (Nagai and Inoue, 2004). This substance contains important proteins with a high content of peptides and essential amino acids, and has high antioxidant properties and free radical scavenging abilities (Guo et al., 2008). 

It has been established that one of the major brain regions involved in epileptogenesis is the hippocampus (Söhl et al., 2000). Therefore, there is a great need to identify mechanisms that cause epilepsy and find new therapeutic targets. Metabolic dysfunction can contribute to seizures and exacerbate associated sequelae such as neuronal loss and cognitive impairment (Pearson-Smith and Patel, 2017). There are several reports suggesting that RJ has neuromodulatory effects, including the improvement of cognitive impairment in mice treated with trimethyltin (Hattori et al., 2011) and the neuroprotective role of RJ shown in a rat model of streptozotocin-induced sporadic Alzheimer's disease (Zamani et al., 2012; Kunugi and Mohammed Ali, 2019). Additionally, the hypothalamic-pituitary-adrenal (HPA) axis is disrupted after temporal and tonic-clonic seizures. Impaired regulation of this axis affects the secretion of luteinizing hormone (LH) and follicle-stimulating hormone (FSH) from the anterior pituitary (Takeda et al., 2016; Aliabadi et al., 2019). Considering that injection of RJ might increase the rate of LH and FSH hormone secretion (Moghaddam et al., 2013), the present study evaluated the possible effects of RJ pretreatment on the LH and FSH hormone levels, as well as hippocampal neurons in a pentylenetetrazole (PTZ)-induced seizure model of ovariectomized (OVX) rats.

## Materials and Methods

### Animals

Fifty-six 10-week-old female Wistar rats weighing 220-250 g were purchased from the Razi Vaccine and Serum Research Institute (Tehran, Iran). The rats were housed and acclimated to laboratory conditions one week prior to experimentation. Rats were kept on a normal light/dark cycle (12:12 light and dark) and temperature (23±2^o^C). Rats had free access to commercially balanced food and water for the duration of the experiment. 

Animals were randomly divided into eight groups (n=7rats/group): 

1- In the control (CTL) group, rats received intraperitoneal (IP) injections of 0.9% saline (10 ml/kg) daily for 15 days. 

2- In the PTZ group, rats received IP injections of 0.9% saline (10 ml/kg) daily for 15 days. Afterward, a single dose of PTZ (80 mg/kg, IP) was injected into the rats (Zendehdel et al., 2015; Momeni et al., 2017). 

3- In the RJ group, rats were administered with RJ (300 mg/kg, IP) daily for 15 days (Karadeniz et al., 2011; Momeni et al., 2017). 

4- In the PTZ-RJ group, rats were pretreated with RJ (300 mg/kg, IP) daily for 15 days. Subsequently, seizure was induced by a single injection of PTZ, 24 hr after the last RJ injection. 

5- In the OVX group, OVX rats received daily injections of normal saline 0.9% for 15 days. 

6- In the PTZ-OVX group, OVX rats received daily injections of normal saline 0.9% for 15 days. Twenty-four hours after the last injection, a single dose of PTZ was administered. 

7- In the OVX-RJ group, OVX rats were treated with RJ for 15 days. 

8- In the PTZ-OVX-RJ group, OVX rats were pre-treated with RJ for 15 days. Twenty-four hours after the last injection, seizure was induced by a single dose of PTZ.

**Figure 1:**
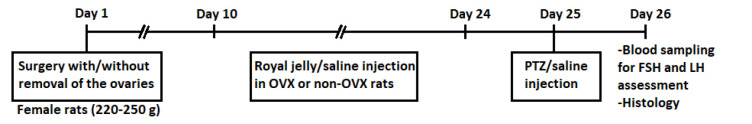
Time line of experimental procedure

It is worth noting that the OVX groups (rats in groups 5-8) underwent surgery to remove their ovaries initially, using the dorsal approach under anesthesia (Ketamine/Xylazine, 80:10 mg/kg, IP). The other groups (rats in groups 1-4) were also anesthetized, but their ovaries were not removed. At the beginning of the experiment, all rats that were subjected to surgery, were not tested until 10 days post-operation (Figure 1). In the PTZ rats, the behavior of rats was monitored for 2 hr. All PTZ-induced seizure rats showed criteria according to previous studies (Erickson et al., 1996; Shafiee et al., 2009). In each group of PTZ-treated rats (PTZ, PTZ-OVX, PTZ-RJ, and PTZ-OVX-RJ groups), 2, 2, 1, and 1 rat died after PTZ injection, respectively, and were replaced by other rats.

### Biochemical assessment

After animal anesthesia with ketamine and xylazine (80:10 mg/kg), blood samples were collected from the rats' hearts. The samples were then placed into tubes containing EDTA-2K and centrifuged at 3000 rpm for 10 min. Serum and plasma samples were stored at -80°C for further analysis. Serum FSH and LH levels were measured using a commercial Enzyme-Linked Immunosorbent Assay (ELISA) kits provided by Monobind Inc. lake forest CA 92630, USA (Cat # 425-300A and Cat # 625-300A, respectively).

### Histology

Animals were given a mixture of ketamine and xylazine. After opening the chest, a cannula was inserted into the left ventricle and secured to the ascending aorta. Solutions were delivered via an incision made in the right atrium. Transfusion was followed by 0.9% saline flush followed by 7% phosphate buffer and 5% formalin flush. The brain was removed from the skull and fixed for 48-72 hr in paraformaldehyde solution.

### Golgi staining

After brain fixation, tissue blocks were placed in 3% potassium dichromate solution for 48 hr in a dark environment. The block was washed with a 0.75% silver nitrate solution and placed in the solution for 72 hr. Tissues were washed with 1% silver nitrate solution. Tissue processing, dehydration counting, clearing and embedding were then performed. Microscopic sections (25 µm) were prepared using a microtome device in the CA1 region of the hippocampus. Dendritic spine counting was performed using the Motic camera and software (Moticam 2000, Spain, and Imaging Tools (version 3)). The number of spines in 5 squares of 2×2 µm in an area of ​​10×10 µm was counted in an image with a magnification of X400. To do this, we counted the number of thorns after fixing the picture and the large square (Hadipour et al., 2020; Hadipour et al., 2021).

### Statistical analysis

Data were analyzed using the Shapiro–Wilk test for checking the normality of data distribution. Three-way ANOVA followed by Bonferroni post-test was used for two effect factors of OVX and RJ treatment in the PTZ and non-PTZ groups (Graph Pad Software version 9.3.1, Inc., San Diego, CA). The significance criterion was set as p<0.05. 

## Results

### Serum LH

Three-way ANOVA revealed significant main effects for PTZ [F(1, 48 = 50.76), p<0.001], OVX [F(1, 48 )= 10.9, p<0.01], and RJ [F(1, 48) = 61.2, p<0.001].

 The interaction between PTZ, OVX and RJ treatments was also significant [F(1, 48 ) = 113.8, p<0.001]. 

The serum LH hormone level in the OVX group was significantly more than the CTL group (p<0.05), while there was no statistically significant difference between the CTL, PTZ-OVX, and PTZ groups. The serum LH hormone level in the PTZ-OVX group was significantly lower than the OVX group (p<0.01).

A significant increase serum LH hormone level was observed in the RJ group compared with the CTL group (p<0.0001), while the LH hormone level in the PTZ-RJ group was not statistically significant difference compared to the PTZ group. The serum LH hormone level in the PTZ-RJ group was significantly lower than the RJ group (p<0.0001).

A significant increase was observed in the LH hormone level of the OVX-RJ and PTZ-OVX-RJ groups compared to the CTL group (p<0.05) and the PTZ group (p<0.001), respectively. Data analysis also showed that the serum LH hormone level in the OVX-RJ group was significantly lower than the LH hormone level in the RJ group (p<0.0001), while the LH hormone in the PTZ-OVX-RJ group was significantly higher (p<0.01) than the PTZ-RJ group. 

**Figure 2:**
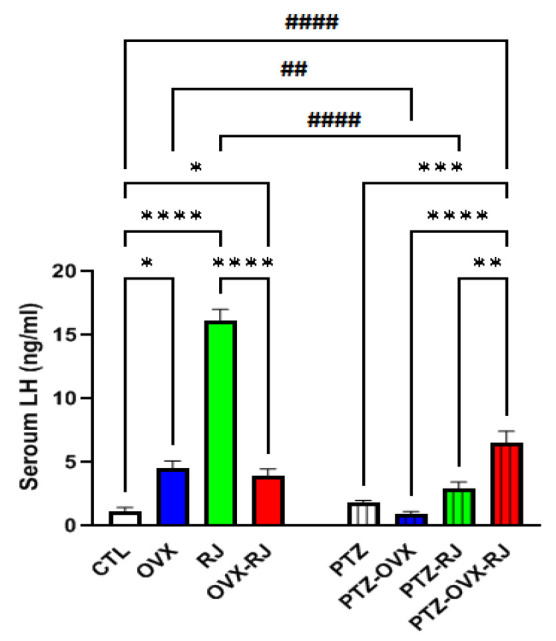
Comparison of serum LH hormone levels among the experimental groups. Data are presented as mean±SEM (*p<0.05, **p<0.01, ***p<0.001, ****p<0.0001,##p<0.01,and####p<0.0001). Abbreviations: CTL; control, PTZ; pentylenetetrazole, RJ; Royal jelly, OVX; ovariectomized (n=7/group).

The serum LH hormone level of the OVX and OVX-RJ groups did not show statistically significant differences, while the serum LH hormone levels of the PTZ-OVX-RJ group were higher than that of the PTZ-OVX group (p<0.0001). The LH hormone levels in the PTZ, PTZ-OVX, and PTZ-OVX-RJ groups were also not statistically significantly different (Figure 2).

### Serum FSH

Concerning serum FSH hormone level, three-way ANOVA revealed significant main effects for PTZ [F(1, 48) = 38.27, p<0.001] and RJ [F(1, 48) = 207.9, p<0.001]. The interaction between PTZ, OVX, and RJ treatments was also significant [F(1, 48 ) = 130.8, p<0.001].

The serum FSH hormone level in the OVX group showed a statistically significant increase compared to the CTL group (p<0.05) while the CTL, PTZ-OVX, and PTZ groups did not exhibit a statistically significant difference. The serum FSH hormone level in the PTZ-OVX group was significantly lower than the OVX group (p<0.05).

A significant increase in the serum FSH hormone level was observed in the RJ group compared to the CTL group (p<0.0001), while the FSH hormone level in the PTZ-RJ group did not show a statistically significant difference compared to the PTZ group. The serum FSH hormone level in the PTZ-RJ group was significantly lower than the RJ group (p<0.0001).

The present results demonstrated a significant increase in the FSH hormone level of the OVX-RJ group (p<0.001) and the PTZ-OVX-RJ group (p<0.0001) compared to the CTL and the PTZ groups, respectively. The serum FSH hormone level in the OVX-RJ group was significantly lower than that of the RJ group (p<0.0001), while the FSH hormone level in the PTZ-OVX-RJ group was significantly higher (p<0.0001) than the PTZ-RJ group. 

**Figure 3:**
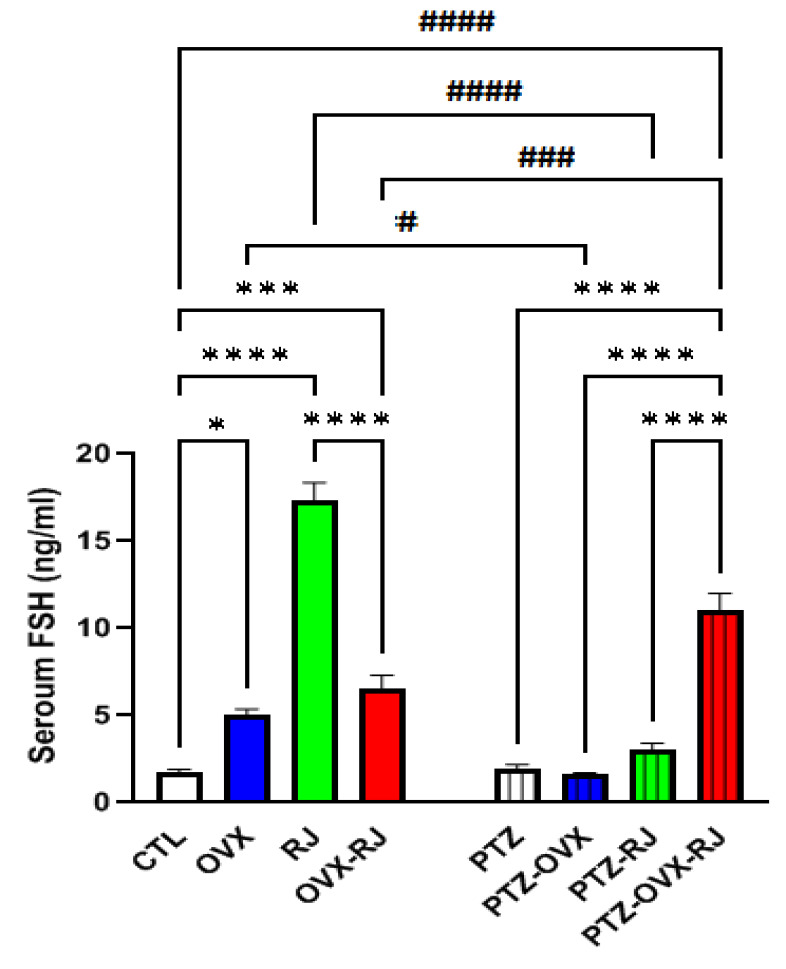
Comparison of serum FSH hormone levels among the experimental groups (n=7/group). Data are presented as mean±SEM (****p<0.0001, ###p<0.001, and ####p<0.0001). Abbreviations: CTL; control, PTZ; pentylenetetrazole, RJ; Royal jelly, OVX; ovariectomized.

Additionally, the serum FSH hormone levels of the OVX, and OVX-RJ groups were not statistically significantly different while the serum FSH hormone levels of the PTZ-OVX-RJ group were higher than that of the PTZ-OVX group (p<0.0001). It was found that the FSH hormone levels in the PTZ, PTZ-OVX, and PTZ-RJ groups were not statistically significantly different (Figure 3). 

### The number of dendritic spines of neurons

**Figure 4:**
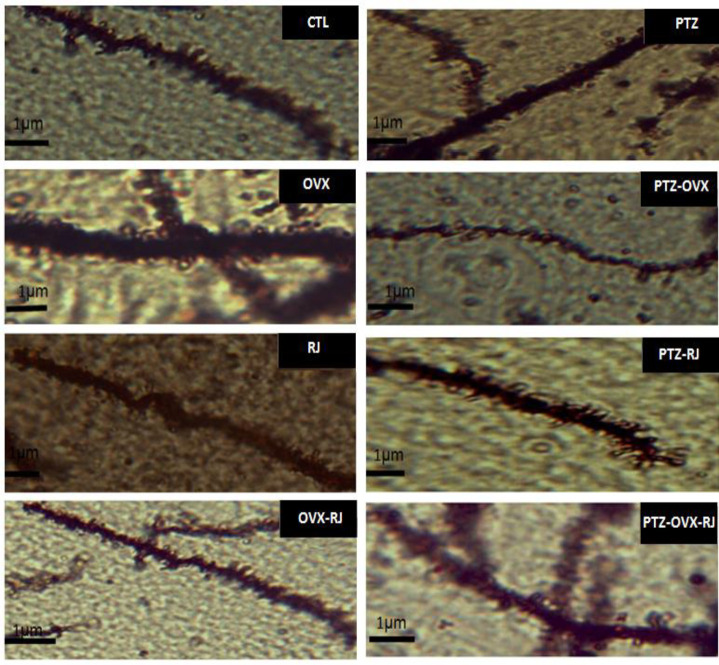
Comparing the experimental groups, regarding micrograph images of the CA1 hippocampus with Golgi staining (magnificationX400). Abbreviations: CTL; control, PTZ; pentylenetetrazole, RJ; Royal jelly, OVX; ovariectomized.

Figure 4 displays micrographs of dendritic spines in the hippocampal CA1 region, stained with Golgi, for each group. The number of neuronal dendritic spines decreased after PTZ injection in both non-OVX and OVX rats.

**Figure 5:**
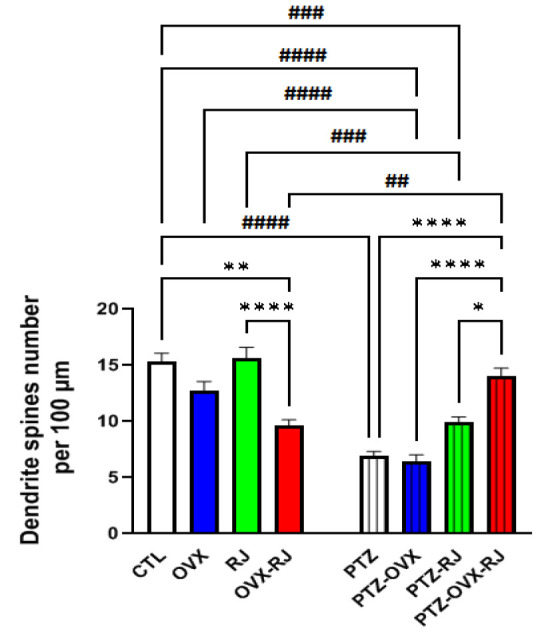
Comparison of the number of hippocampal CA1 dendritic spines among experimental groups (n=7/group). Data are presented as mean±SEM (*p<0.05, ***p<0.001, ****p<0.0001, ##p<0.01, ###p<0.001, and ####p<0.0001). Abbreviations: CTL; control, PTZ; pentylenetetrazole, RJ; Royal jelly, OVX; ovariectomized.

To quantify the number of dendritic spines (Figure 5), a three-way ANOVA was conducted. The results showed a significant main effect for PTZ [F(1, 48) = 50.7, p<0.0001], OVX [F(1, 48) = 4.6, p < 0.05], and RJ treatment [F(1, 48) = 11.8, p < 0.001]. The interaction among PTZ, OVX, and RJ treatment was also significant [F(1, 48) = 12.7, p < 0.0001].

It was found that the number of dendritic spines in the PTZ, PTZ-OVX, and PTZ-RJ groups was significantly lower than that in the CTL (p<0.0001), OVX (p<0.0001), and RJ groups (p<0.001), respectively. However, there was a significant increase in the number of dendritic spines in the PTZ-OVX-RJ group compared to the OVX-RJ group (p<0.01). Importantly, there was no statistically significant difference in neuron numbers between the PTZ-OVX-RJ and CTL groups.

Further analysis revealed that, there was no significant difference in the number of dendritic spines between the OVX and CTL groups. Also, there was no significant difference in the number of dendritic spines between the PTZ-OVX and PTZ groups.

The number of dendritic spines was not significantly different between the RJ and CTL groups. Likewise, in the PTZ conditions, there was no significant difference in the number of dendritic spines between the PTZ-RJ and PTZ groups.

However, a significant decrease in the number of dendritic spines was observed in the OVX-RJ group compared to the CTL group (p<0.0001). Also, the number of dendritic spines in the PTZ-OVX-RJ group was increased compared to the PTZ, PTZ-OVX, and PTZ-RJ groups (p<0.0001, p<0.0001, and p<0.05).

## Discussion

Recent research has demonstrated that PTZ can cause biochemical and neuronal changes in the hippocampus of epileptic animals (Vasilev et al., 2018). In addition, PTZ remains effective from 3 hr post-injection until at least 1 week after PTZ injection (Vasilev et al., 2018). Our results also showed that PTZ decreased the neuronal dendritic spines, as well as the levels of LH and FSH in PTZ-OVX rats compared to the controls (i.e. OVX group). This finding is consistent with a classical study that demonstrate reduced concentrations of FSH and LH in OVX rats with acute or chronic epilepsy (Bhanot and Wilkinson, 1982). Another previous study also showed that PTZ can decrease the number of neurons in the hippocampus of PTZ-induced rats (Momeni et al., 2017). Menopause is a physiological condition that triggers encephalitis, leading to the production of cytokines such as interleukin-1β (IL-1β), IL-1α, and IL-6. Estrogen inhibits the expression of inflammatory cytokines, thereby causing seizures (Yasui et al., 2006). Pro-inflammatory cytokines like IL-1β, IL-2, and IL-6 are typically found at low levels in the brain, but their levels increase following a stroke (Scorza et al., 2018). Peripheral inflammation can damage the blood-brain barrier, potentially inducing or worsening epileptogenesis (Rivest et al., 2000; Riazi et al., 2010). Controlling inflammation in these disorders may thus decrease the epilepsy risk.

Previous research has demonstrated that the seizure pattern changes due to decreased levels of beta-estradiol and progesterone during menopause or ovarian surgery (Aliabadi et al., 2019). In an animal model of PTZ-induced seizures, it was observed that both acute and chronic estrogen administration reduced seizure onset time and susceptibility (Mohammadpour et al., 2012; Ebrahimzadeh-Bideskan et al., 2018). Conversely, progesterone was found to increase latency to seizures (Edwards et al., 1999; Verrotti et al., 2007). Long-term administration of estrogen and estrogenic compounds like soy extract has been shown to protect hippocampal neurons in PTZ-induced rats (Ebrahimzadeh-Bideskan et al., 2018). The difference in hippocampal dendritic spine numbers between the PTZ and PTZ-OVX groups was not linked to LH and FSH hormone levels or dendritic spine count. This suggests that factors other than hormone levels, such as estrogen and other steroid hormones, nitric oxide levels, brain-derived neurotrophic factor, etc., may have played a role (Momeni et al., 2017).

In addition, unlike our study, it has been demonstrated that individuals with epileptic disorders exhibit HPA axis impairment, which leads to an increased gonadotropin-releasing hormone (GnRH) pulse rate and secretion of LH and FSH hormones (Hamed, 2016; Aliabadi et al., 2019). In the current study, it was also observed that in the OVX group, the dendritic spine count of hippocampal neurons decreased, while the levels of LH and FSH hormones increased. Given the inhibitory impact of serum pituitary hormones LH and FSH on ovarian estrogen and progesterone secretion levels, the reduction in dendritic spine count of hippocampal neurons can be rationalized (Sales et al., 2010; Bayer and Hausmann, 2011).

While the main significant finding of this study was that RJ exposure increases the serum levels of LH and FSH of the RJ group but it did not lead to notable changes in neuronal dendritic spines in the RJ group. Consistent with our findings, previous research has reported that RJ injection can elevate the secretion rate of LH and FSH hormones (Moghaddam et al., 2013). Al-Sanafi et al. (2007) reported no significant changes in FSH levels, but they observed an increase in LH levels following treatment with RJ. RJ contains acetylcholine (1 mg/g) (Al-Sanafi et al., 2007). Acetylcholine may stimulate the secretion of human chorionic gonadotropin at the hypothalamic level, leading to an increase in FSH and LH (Kornya et al., 2001).

Studies have shown that RJ supplements can enhance estrogen synthesis and maintain low levels of FSH and LH in the bloodstream, primarily due to the presence of fatty acids, particularly 10-hydroxyl-2-decenoic acid (Imai et al., 2012). Based on this, it is assumed that one of the goals of the RJ supplement is to enhance the quality of life during the post-menopausal phase (Sharif and Darsareh, 2019). RJ is known to contain hormones like progesterone, prolactin, estradiol, and insulin-like growth factor-1 (IGF-1), which suggests its potential as an endocrine disruptor (Suzuki et al., 2008). High levels of IGF-1, as found in RJ, may sensitize pituitary cells to GnRH. Previous studies have shown that incubation with IGF-1 for 2-3 days sensitized rainbow trout pituitary cells to GnRH (Weil et al., 1999). Therefore, it can be hypothesized that RJ increases LH and FSH levels through its IGF-1 factor which sensitizes pituitary cells to GnRH and contributes to its estrogenic activity. Additionally, Mishima et al. (2005) reported that RJ exerts estrogenic effects by interacting with estrogen receptors and causing changes in gene expression and cell function (Mishima et al., 2005). These findings suggest potential mechanisms underlying the estrogenic activity of RJ in this study. 

In the PTZ condition, RJ exposure did not change the serum levels of LH and FSH in the PTZ-RJ rats compared to the PTZ group. The neuronal dendritic spines of the PTZ-RJ rats increased insignificantly compared to the PTZ group but they were significantly decreased compared to the CTL group. Here, a helpful explanation of RJ's effects on the hippocampal neurons in PTZ-RJ rats may be provided. However, it has been demonstrated that PTZ acts as a γ-aminobutyric acid type A receptor antagonist (Huang et al., 2001) and induces hyperactivation of hippocampal neurons in an N-methyl-D-aspartate (NMDA) receptor-dependent manner (Zaitsev et al., 2015), resulting in neuronal apoptosis (Li et al., 2021). 

Under non-PTZ condition, it has been noted that RJ decreased significantly the neuronal dendritic spines in OVX-RJ rats compared to the CTL group. However, FSH and LH hormones levels were significantly increased in the OVX-RJ group compared to the normal female rats of the CTL group. Recent studies also found that RJ did not alter neuron count or dendritic spines in normal male rat frontal cortex neurons (Jalili et al., 2019) and hippocampus (Momeni et al., 2017), which aligns with our findings. In contrast, it has been reported that RJ promotes the differentiation of various brain cells such as neurons, astrocytes, and oligodendrocytes, and increases the formation of neurons (Hattori et al., 2007). Hashimoto et al. (2011) demonstrated that oral administration of RJ promoted the expression of neurotrophic factors mRNA in glial cell lines in the hippocampus of adult mice brains (Hashimoto et al., 2005). This contradicts the results of our study. The discrepancy may be due to differences in the dosage of RJ and evaluation methods. While the current study revealed normal levels of LH and FSH, we expected that RJ treatment in the OVX-RJ group could have neurogenic and tropism effects due to its compounds such as nucleotides (guanosine, adenosine, and uridine) and phosphates (adenosine monophosphate, adenosine diphosphate, and adenosine triphosphate), which aid in normal neuronal growth (Balan et al., 2020). 

Under PTZ condition, in the current study, a remarkable increase in the neuronal dendritic spines was observed in the PTZ-OVX-RJ rats. Furthermore, RJ increased serum levels of LH and FSH in the OVX-RJ rat seizure model. Therefore, RJ may have beneficial effects against the detrimental consequences of estrogen deficiency and epilepsy (Mishima et al., 2005; Yasui et al., 2006), which lead to the degeneration of hippocampal neurons in OVX rats exposed to PTZ. Although sudden withdrawal of estrogen affects the autonomic nervous system, it can lead to the development of neurodegenerative diseases (Balan et al., 2020), interestingly, FSH blockade has been shown to improve cognition in mice with Alzheimer's disease (Xiong et al., 2022), and genetic ablation of LH receptors has been found to reduce amyloid pathology in mice with Alzheimer's pathology (Lin et al., 2010). These findings suggest that, both gonadotropins may exacerbate the progression of Alzheimer's disease. However, it was challenging to precisely determine whether RJ could rescue hippocampal neurons from PTZ-induced apoptosis in OVX rats. Therefore, we propose evaluating the effects of RJ on the expression levels of Bcl-2 and/or Bax in hippocampal neurons in the OVX rat epilepsy model.

In conclusion, this study demonstrated that RJ enhances LH and FSH levels, as well as the dendritic spines in the hippocampus of PTZ-OVX rat models. However, the study does not provide evidence for RJ's ability to promote neurogenesis *in vivo*, such as through BrdU labeling. Therefore, RJ may as a useful supplementary nourishment has adequate security against the ruinous impacts of estrogen insufficiency conjointly epilepsy which cause degenerated hippocampus neurons in OVX rats exposed to PTZ. Further studies are needed to confirm that RJ is an effective dietary supplement for improving the quality of life of postmenopausal women.
